# Analysis of gut microbiota profiles and microbe-disease associations in children with autism spectrum disorders in China

**DOI:** 10.1038/s41598-018-32219-2

**Published:** 2018-09-18

**Authors:** Mengxiang Zhang, Wei Ma, Juan Zhang, Yi He, Juan Wang

**Affiliations:** 1 Department of Biomedical Informatics, School of Basic Medical Sciences, Peking University, Beijing, 100191 China; 2 Autism Research Center, Peking University Health Science Center, Beijing, 100191 China; 3 Central Laboratory, Navy General Hospital of PLA, Beijing, 100191 China; 4 Department of pediatrics, Peking University Third Hospital, Beijing, 100191 China

## Abstract

Autism spectrum disorder (ASD) is a set of complex neurodevelopmental disorders. Recent studies reported that children with ASD have altered gut microbiota profiles compared with typical development (TD) children. However, few studies on gut bacteria of children with ASD have been conducted in China. Here, in order to elucidate changes of fecal microbiota in children with ASD, 16S rRNA sequencing was conducted and the 16S rRNA (V3-V4) gene tags were amplified. We investigated differences in fecal microbiota between 35 children with ASD and 6 TD children. At the phylum level, the fecal microbiota of ASD group indicated a significant increase of the *Bacteroidetes/Firmicutes* ratio. At the genus level, we found that the relative abundance of *Sutterella*, *Odoribacter and Butyricimonas* was much more abundant in the ASD group whereas the abundance of *Veillonella* and *Streptococcus* was decreased significantly compared to the control group. Functional analysis demonstrated that butyrate and lactate producers were less abundant in the ASD group. In addition, we downloaded the association data set of microbe–disease from human microbe–disease association database and constructed a human disease network including ASD using our gut microbiome results. In this microbe–disease network based on microbe similarity of diseases, we found that ASD is positively correlated with periodontal, negatively related to type 1 diabetes. Therefore, these results suggest that microbe-based disease analysis is able to predict novel connection between ASD and other diseases and may play a role in revealing the pathogenesis of ASD.

## Introduction

Autism spectrum disorder (ASD) refers to a group of complex neurodevelopmental disorders with early life stage, characterized by deficits in social communication and by restricted and fixated behavior. A meta-analysis of the public health and primary care centers in the UK estimated that the prevalence of autism was 26.6 per 10,000 in mainland, based on eighteen epidemiological studies^1^. It is now well accepted that both genetic and environment factors are related to the aetiology of ASD^2^. Common non-neurological symptoms in ASD patients are gastrointestinal (GI) disorders^3^. Rigid-compulsive behaviors, unusual sleeping or eating habits and oppositional behavior were associated with GI disorders^4,5^.

Emerging evidence suggests that various diseases are associated with altered gut microbiota, including ASD, schizophrenia, depression and Parkinson’s disease^6^. The gut microbiota may control the central nervous system (CNS) through gut-brain axis^7^. The gut-brain axis consists of bidirectional communication between the central and the enteric nervous system, linking emotional and cognitive centers of the brain with peripheral intestinal functions^8^. The mechanisms of the bidirectional communication between microbiota and gut-brain axis, including neural, immune, endocrine and metabolic pathways, are complicated^9–11^. GI symptoms have a strong association with mitochondrial disease and a large subgroup of individuals with ASD demonstrate abnormalities in mitochondrial function as well as GI symptoms^12^. Gut microbiota with its metabolites and components can impact the host physiology, disrupt mitochondrial function, adjusting gut barrier function, energy homeostasis, mucosal inflammation and behavior^13–15^. Changes in the microbiota and their metabolic end products could lead to abnormal redox and mitochondrial metabolism, as well as immune dysfunction. It has been hypothesized that disruptions in the microbiome may be involved in etiology and/or pathophysiology of ASD^16^. *Clostridiales* and *Bacteroidetes/Firmicutes* ratios were increased in ASD children with functional gastrointestinal disorders^17^. Some clinical studies revealed that patients with ASD had increased inflammation^18,19^, immune system dysfunction^20,21^, mitochondrial disorders^22^ altered metabolic capacity^23,24^, GI disorders, seizures^25^ and gut microbial disorders^26–28^.

One research on ASD children in Slovakian showed increases in the amount of *Lactobacillus spp* and *Desulfovibrio spp* and a significant reduction of the *Bacteroidetes/Firmicutes* ratio in the fecal microbiota^26^. Another study on regressive autism also showed that *Desulfovibrio* was more common in autistic subjects than in controls^29^. Analysis of real-time PCR data indicated that mean counts of *Clostridium clusters I* and *XI* in ASD children were greater than those in TD children^30^. Conversely, ASD children possessed lesser amounts of *Bifidobacterium* than TD children^31^. *Bifidobacterium* are associated with the biosynthesis and cellular content of folate^32^. The decreased abundance in *Bifidobacteria* potentially leaded to reduced folate production by microbiota in individuals with ASD. ASD has been related to abnormalities in folate metabolism and folate pathway abnormalities may be a major metabolic disturbance in ASD^33^. These studies have emphasized that alterations in the composition of the gut microbiota have been implicated in ASD.

However, most studies of the gut microbiota in patients with ASD have been focused on Western populations, it is important to expand these studies to non-Western diet populations in order to fully understand the range of variation of the gut microbiota in patients with ASD and how gut microbes affect the pathogenesis of ASD. Thus, we performed 16S rRNA sequencing of stool samples from 40 children with ASD and 7 TD children in China, aiming to elucidate changes in fecal microbiota in children with ASD and find the effects of microbiota changes on metabolism. In addition, we downloaded the association data set of microbe–disease from human microbe–disease association database (HMDAD) and constructed a microbe-based human disease network(HMDN) including ASD based on microbiome similarity^34^.

## Results

### Subject characteristics

A total of 40 children with ASD and 7 TD children were initially recruited. Five of the 40 children with ASD and one of the 7 TD children had low quality reads. Therefore, 35 children with ASD and 6 TD children were included in the final analysis. All children were between 3 to 8 years old, with a mean (±SD) age of 4.9 (±1.5) years for the ASD group and 4.6(±1.1) years for the control group (Table 1).

**Table 1:** Summary of subject characteristics.

	ASD group	Control group
Total participants	35	6
Age(years)	4.9 ± 1.5^#^	4.6 ± 1.1
Male/Female	29/6	5/1
Constipation	11	0
Diarrhea	2	0
abdominal distention	8	0

^#^All values are mean ± standard deviation.

### Microbiota changes in children with ASD

**Figure 1:**
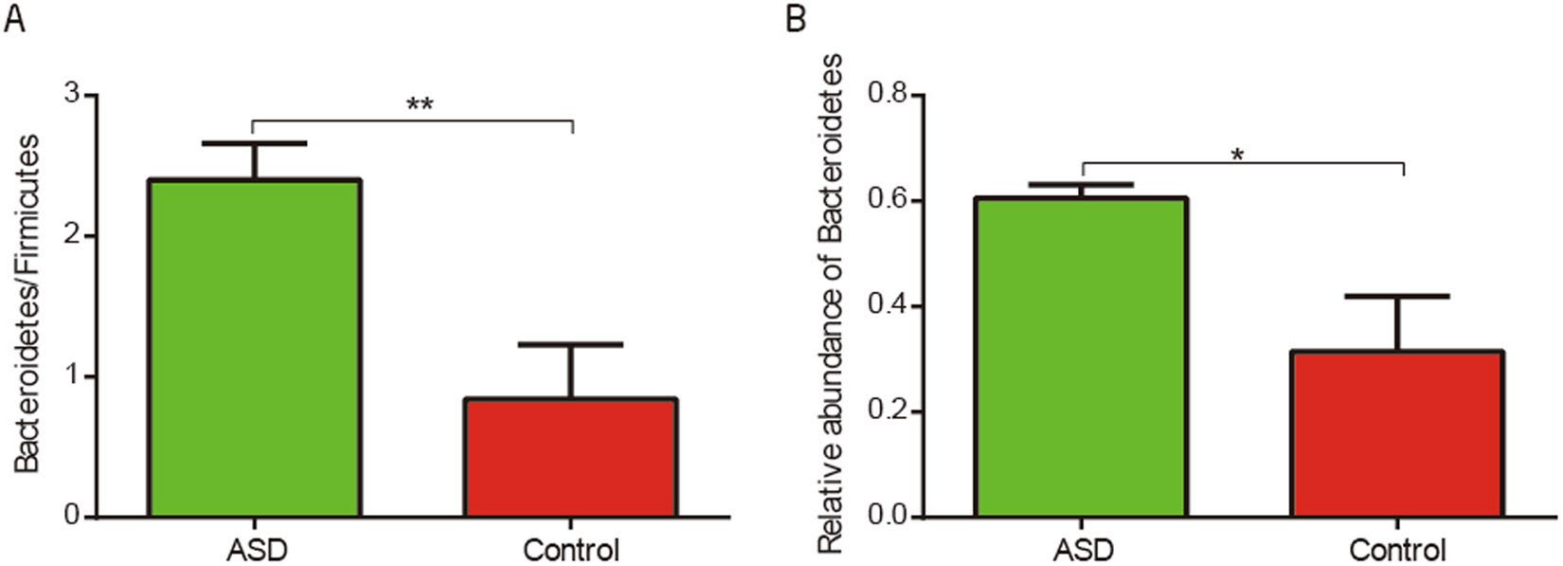
(**A**) Comparison of the ratio of *Bacteroidetes/Firmicutes* between ASD children and typical development children (**p < 0.005, Wilcoxon rank-sum test). (**B**) Box plot representation of the relative abundance of *Bacteroidetes* (*FDR-corrected p < 0.05, Wilcoxon rank-sum test). The boxes represent the mean ± Standard Error of Mean (SEM).

**Figure 2:**
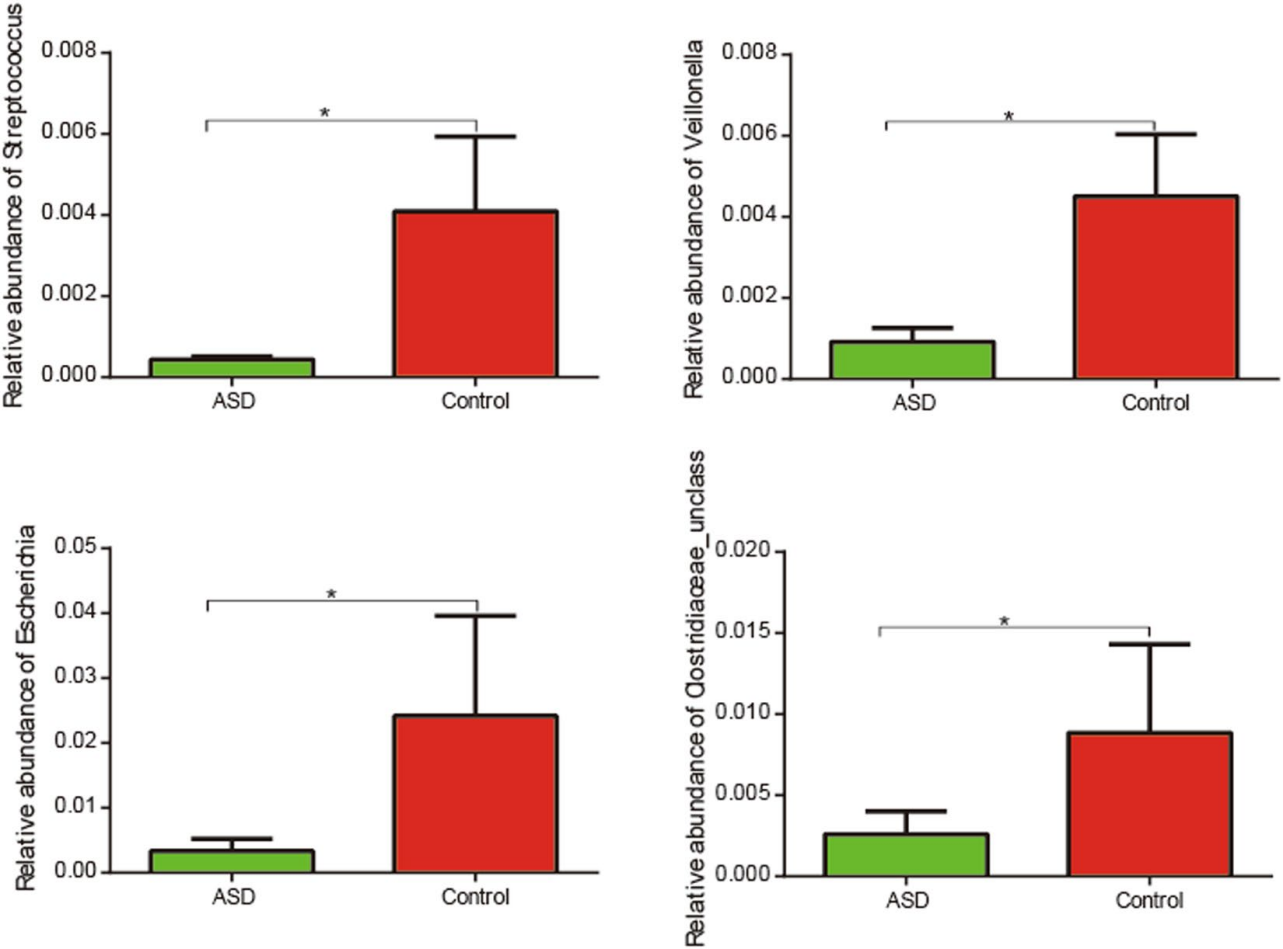
Box plot representation of the relative abundances of bacterial genera that significantly decreased in ASD group compared with control group. The boxes represent the mean ± SEM, *FDR-corrected p < 0.05, Wilcoxon rank-sum test.

Our study discovered the difference in the fecal microbiota between ASD and control groups. Intestinal flora imbalance of children with ASD was mainly represented by a different bacterial abundance at the level of phylum, as well as at the level of genus (Table 2, Fig. S1). At the level of phylum, the ratio of *Bacteroidetes/Firmicutes* (p < 0.05, Wilcoxon rank-sum test; Fig. 1a.) was significantly higher in the ASD group compared to the control group due to a significant increase of the relative abundance of *Bacteroidetes* (FDR-corrected p < 0.05, Wilcoxon rank-sum test; Fig. 1b). Genus level analysis showed *Bacteroides*, *Faecalibacterium*, *Lachnospiraceae_unclass* and *Oscillospira* were abundant in both ASD and control subjects (Supporting Dataset S1). We observed that *Streptococcus*, *Veillonella* and *Escherichia* were was significantly less abundant in ASD group compared to control group (Fig. 2).

**Table 2:** The bacterial abundance at the level of phylum and genus in two groups with significant difference (q-value < 0.05).

	ASD (mean)	Control(mean)	p-value	q-value
**phylum level**				
p__Bacteroidetes	0.6057	0.3149	0.00586	0.03246
p__Firmicutes	0.3349	0.6299	0.01279	0.03246
p__Bacteria_unclass	0.0142	0.1866	0.01623	0.03246
**genus level**				
g__Streptococcus	0.000437	0.024549	0.00043	0.01576
g__Veillonella	0.000919	0.027029	0.00182	0.03097
g__Escherichia	0.003306	0.024151	0.00330	0.04700
g__Clostridiaceae_unclass	0.002599	0.053084	0.00297	0.04567
g__Actinomyces	0.000009	0.00009	2.76E + 01	0.00467
g__Parvimonas	0.00009	0.00830	0.001832	0.03097
g__Bulleidia	0.000002	0.000014	0.001432	0.03024
g__Peptoniphilus	0	0.000011	0.000653	0.01576

**Figure 3:**
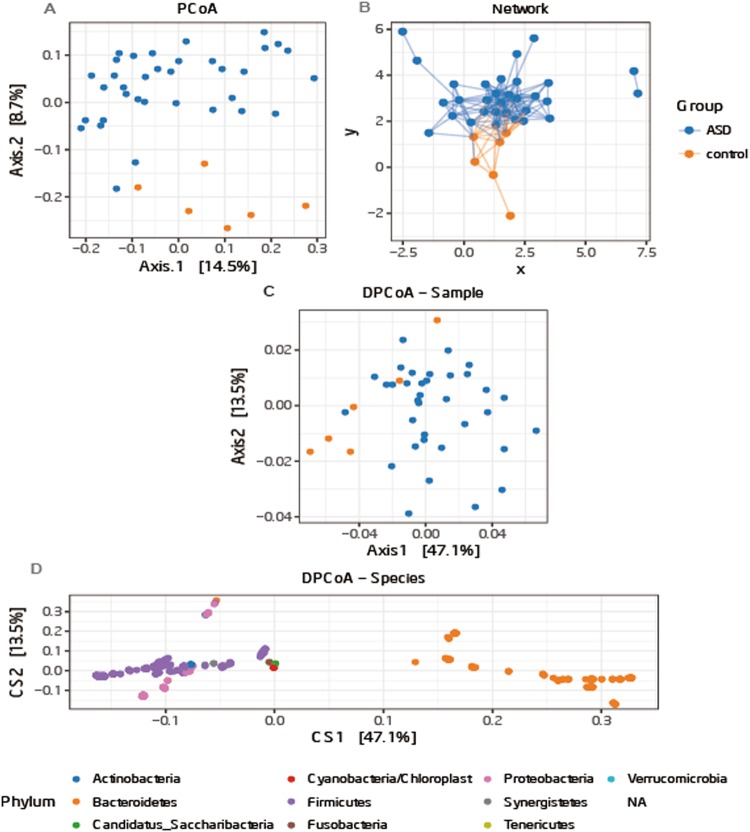
(**A**) PCoA of bacterial beta diversity based on the Bray-Curtis dissimilarity. ASD and control subjects are coloured in blue and orange, respectively. (**B)** Network of ASD and control group based on Bray-Curtis dissimilarity; (**C**) community and (**D**) species points for DPCoA.

Shannon index which reflects the alpha diversity revealed no significant differences between ASD and control groups, but the diversity of most of the children with ASD was lower than that of TD children (Fig. S2). Analysis of the beta diversity calculated on the Bray-Curtis dissimilarity and the network revealed that the bacterial microbiota of ASD group clusters apart from that of control group (Fig. 3a,b, Permutation test, p = 0.002). Analysis of DPCoA showed that the differences between gut microbiota in the two groups were *Bacteroidetes* at the phylum level (Fig. 3c,d).

### Functional analysis

**Figure 4:**
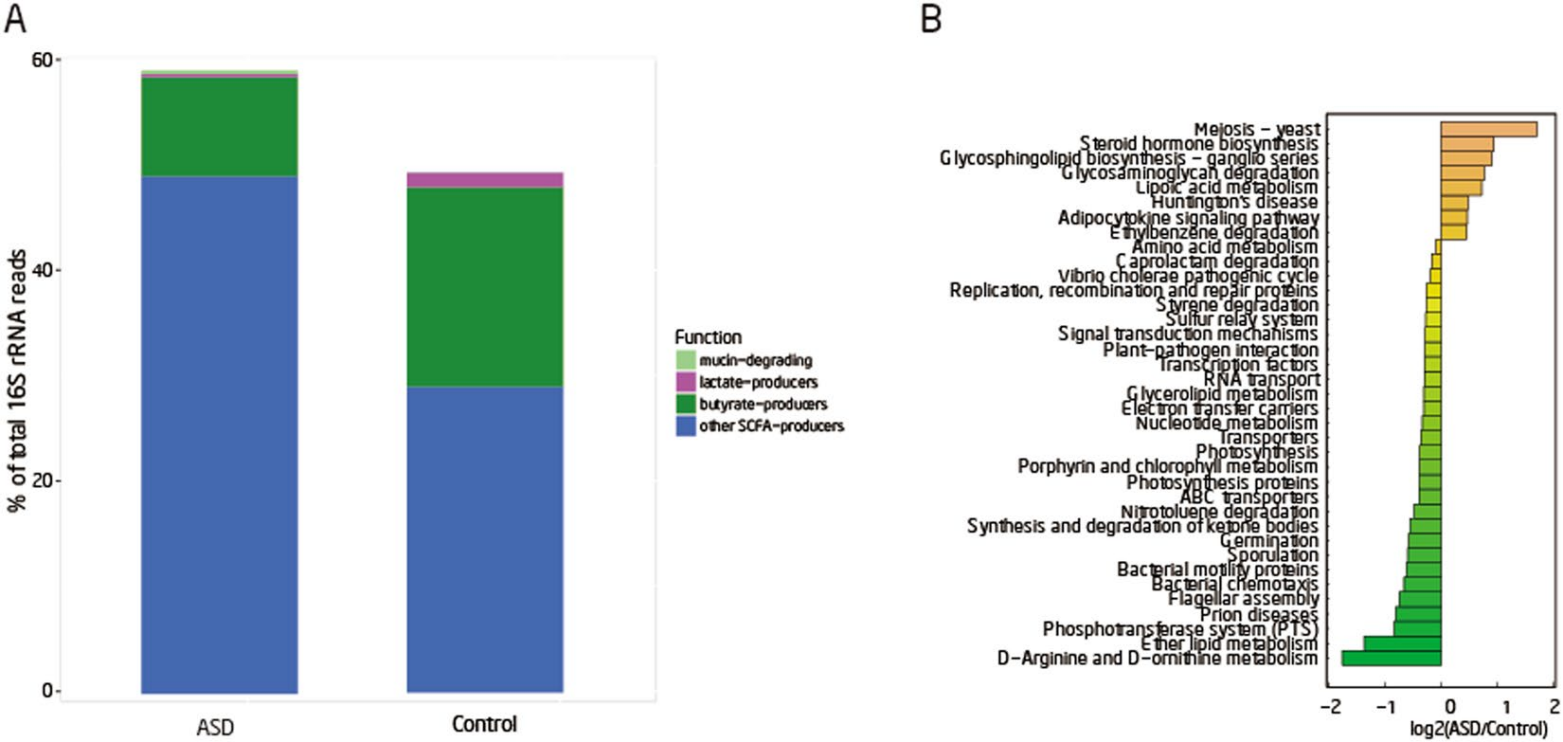
(**A**) Mean proportion of four functional groups between ASD and control. The genera depicted are known to degrade mucin and produce lactate, butyrate, or other short chain fatty acids (SCFA) such as propionate, succinate, or acetate. (**B**) Thirty-seven known functions that differ significantly between cases and controls (p < 0.05) as determined by the log of the ration between ASDs and controls. Twenty-nine of these functions are the highest in ASDs relative to controls while eight are the highest in controls relative to ASDs.

In this study, we compared the differences of four groups of functional bacteria of short chain fatty acids (SCFA) producers between ASD and control groups. We found that butyrate and lactate producers were more abundant in the control group while mucin-degraders and other SCFA-producers were more abundant in the ASD group (Fig. 4a) although these differences of relative abundance were not statistically significant. Butyric acid can promote the synthesis of mucin^35^ and enhance intestinal tight junction integrity^36^. The lower relative abundances of mucolytic *Akkermansia muciniphila* bacterium in children with autism caused mucus barrier changes^37^. A high percentage of abnormal intestinal permeability has been reported in patients with autism^38^. Research stated that the genera *Fusobacterium, Eubacterium, Anaerostipes, Subdoligranulum, Faecalibacterium and Roseburia* could produce butyrate while *Lactoabcillus, Bifidobacterium, Streptococcus, and Lactococcus* produce lactate and the genera *Prevotella and Akkermansia* could produce mucin^39^. In our study, *Faecalibacterium* and *Roseburia* was decreased in the ASD group compared with the control group although this difference of relative abundance was not supported by the statistical analysis. The alteration of bacteria producing butyric acid may be related to the abnormally-elevated intestinal permeability in ASD patients.

In our study, we investigated 254 KEGG pathways.The result of KEGG pathways analysis showed that D−Arginine and D−ornithine metabolism, ether lipid metabolism, bacterial chemotaxis, neurodegenerative diseases -prion diseases, phosphotransferase system (PTS) and flagellar assembly genes were more abundant in ASD group than that of control group while meiosis-yeast, steroid hormone biosynthesis, glycosaminoglycan degradation and lipoic acid metabolism were enriched in the control group (p value < 0.05, Wilcoxon rank-sum test, Fig. 4b, Supporting Dataset S2). PTS system, which was enriched in ASD is also related to diabetes^40^. However, after FDR correction, no pathways were significantly different between two groups.

### Construction of the HMDN including ASD

**Figure 5:**
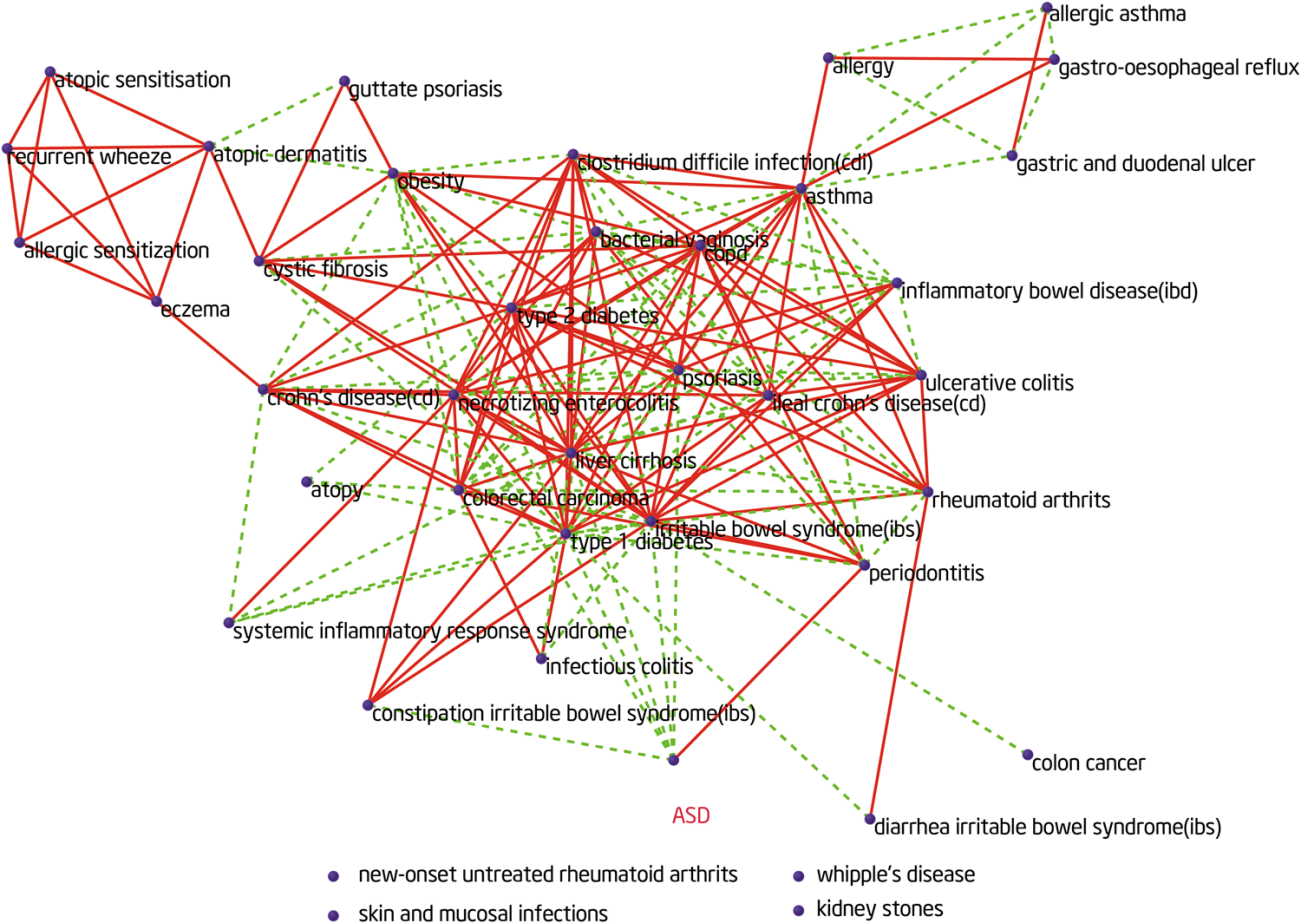
The human microbe-based disease network (HMDN) including ASD. In the network, each node represents one disease. The green dotted lines or the red solid lines represent a negative or positive microbe-based link between two diseases, respectively.

**Figure 6:**
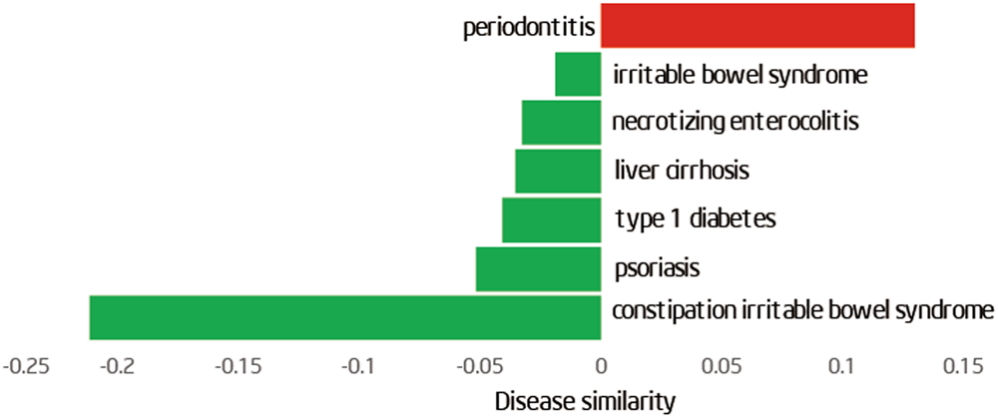
Microbiota changes between ASD and the diseases in the microbe-based disease network. Height of the green (red) bars represents the strength of negative (positive) similarity between ASD and corresponding diseases.

Based on the human microbe–disease association database (HMDAD)^34^,we calculated the microbe similarity between ASD and other diseases. We constructed a network (Fig. 5) with a total 188 links (links represent microbe similarities between diseases), including 114 positive links (consistent changes in microbiome) and 74 negative links (reversed changes in microbiome) among 40 diseases. In this network, each node represents one disease. The red solid lines or the green dotted lines represent a positive or negative microbe-based link between two diseases, respectively. We found that ASD and periodontal have similar microbiota changes. In contrast, ASD and type 1 diabetes, constipation irritable bowel syndrome, psoriasis have opposite microbiota changes (Fig. 6). However, the correlation of these diseases was unclear. Clinical evidence will be needed to verify our finding in the future.

## Discussion

The frequent occurrence of GI issues in ASD patients imply the possible involvement of the gut microbiota in gastrointestinal pathophysiology of ASD. Thus, it is important to detect the change of gut microbiota in ASD children. Our results demonstrated that the ratio of *Bacteroidetes/Firmicutes* was significantly higher in children with ASD emanating from the higher abundance of *Bacteroidetes* phylum. Inconsistently with other observations, a decreased *Bacteroidetes/Firmicutes* ratio has been reported in subjects with autism due to a significant reduction of *Bacteroidetes* in these individuals^26,41^. Gut microbiota of children in a rural African village in Burkina Faso showed an increase in *Bacteroidetes* and a decrease in *Firmicutes* relative to European children^38^. The decreased *Bacteroidetes/Firmicutes* ratio in Slovakia and Italy population and the increased *Bacteroidetes/Firmicutes* ratio in Chinese population may be due to different living environment and eating habits. Western diet habits may affect the composition of the gut microbiota^42^. Most studies of the gut microbiota in patients with ASD have been focused on Western populations, it is important to expand these studies to non-Western diet populations in order to fully understand the range of variation of the gut microbiota in patients with ASD and how gut microbes affect the pathogenesis of ASD. Furthermore, we discovered that the abundance of *Veillonella* was significantly decreased in stools of children with ASD. Consistently with this observation, the decrease of *Veillonella* has been reported also in subjects with autism^41^. *Veillonella* can ferment lactate^43^, indicating that the decreased *Veillonella* may disturb the fermentation of lactate in children with ASD. Our study also suggested *Streptococcus* and *Escherichia* were depleted in ASD subjects in comparison to control subjects, in line with the results obtained in previous study^44^.

As the ‘second genome’ of human beings^45^, gut microbiota play important roles in human health and diseases. Carbohydrates are essential sources of energy for human beings and microbiota, but our own enzymes can’t degrade most complex polysaccharides and plant cellulose. These indigestible carbohydrates can be fermented by microbiota in the gut and produce energy related products (such as SCFA, mainly including acetic acid, propionic acid and butyric acid). SCFAs are plausibly linked to ASD and can induce widespread effects on gut, brain and behavior^46^. Butyrate is recognized as an anti-inflammatory SCFA that contributes to colon health^47,48^. Research showed that butyrate could rescue ASD cells during oxidative stress and enhance mitochondrial function in the context of physiological stress^49^. Butyrate can modulate neurotransmitter gene expression^50^ and the ASD-related genes in cell line models^51^. We found that butyrate and lactate producers were less abundant in stools of children with ASD, although the difference was not statistically significant in this small study. It indicated that the altered gut microbiota in ASD children could influence the production of SCFA and then disturb intestinal health.

The KEGG database is an important functional database that is used to annotate genes^52^. Genes can be projected into the KEGG PATHWAY database reveal interactions with other genes that may influence the health of the host^53^. The 16 Sr RNA mining of our data showed that some genes were much more abundant in ASD group than that of control group. Major function category including amino acid metabolism, bacterial chemotaxis, RNA transport and porphyrin and chlorophyll metabolism genes was much more abundant in terms of the % of total reads in control group than that of ASD group. Lipoic acid metabolism and Glycosphingolipid biosynthesis was much more abundant in ASD group (supporting dataset S2). The above differences in gene abundances were not statistically significant, but are worthy of further investigation.

An important discovery in the study is that the microbe-based similarity between ASD and periodontitis is positive, indicating that the changes of the shared microbes tend to be similar in the two diseases. Children with ASD had significantly higher periodontal treatment needs compared with unaffected controls^54^. In addition to, autistic children had higher dental caries prevalence than that in their unaffected peers^55^. This finding further presents a potential reason or mechanism by which ASD is positively correlated with periodontitis. Strikingly, type 1 diabetes shows a negative microbe-based similarity with ASD, indicating that ASD is negatively correlated with type 1 diabetes in their shared microbiota. For example, *Veillonella* is decreased in ASD but increased in type 1 diabetes^56^. However, the exact relationship between type 1 diabetes and ASD is complex and deserves further study.

Our discoveries add to available clinical evidence that altered gut microbiota could be linked to the development of ASD. More importantly, we found that microbe-based disease analysis could predict novel connection between ASD and other diseases; it may offer a promising prospect for revealing the pathogenesis of ASD. In the future, we can explore the pathogenesis of autism and divide ASD into different subtypes based on alteration of gut microbiota and microbe-based disease analysis.

However, there is limitation exist in our study. First, the sample size of controls in this study is small. Because gut microbiota is closely related to ASD, our results may be significant despite that limitation. Second, the HMDAD we used has not been updated since it was established. Hence, the available microbe–disease associations only represent a small number of microorganisms and human diseases before the date (July 2014).

## Subjects and Methods

### Participants

All children with ASD participating in this study were diagnosed with autistic spectrum disorder according to DSM-5 (Diagnostic and Statistical Manual of Mental Disorders-5th Edition). They were enrolled from one local family fraternity group, a group of unrelated autistic families. Children who had fragile X syndrome, tuberous sclerosis, significant sensory impairment, clinically evident inflammatory conditions, coeliac disease, special diet (such as ketogenic diet) and brain anomalies detected by magnetic resonance imaging were excluded.

Control group children were recruited from two kindergartens through pediatricians. Children who had psychiatric conditions (such as depressive disorder, schizophrenia and bipolar disorder) were excluded according to their medical examinations for enrollment and parent interview. All subjects did not take antibiotics, antipsychotics, probiotics and prebiotics in the past month prior to the sample collection.

At last, 40 children with ASD and 7 typically developing children were enrolled in Beijing. The ages of autistic children and control children were 3 to 8 years. This study has been approved by the Institutional Review Board of Peking University (Ethical Review Document No: IRB00001052-16059). We only use stool samples to analyze the related genes of the microbiota. The study group gained informed consent from the parents/guardians for the collection of stool samples and trial information. We confirmed that all methods were performed in accordance with relevant guidelines and regulations.

### Stool collection

Fecal specimens were collected in the homes of the participants by their parents. Immediately deep freezing was required to preserve the specimens, and then fecal specimens were shipped to Medical informatics laboratory in Peking University Health Science Center on the same day where each specimen was frozen at −80 °C until DNA extraction.

### Library preparation and Illumina sequencing

The mixture of purified PCR products was generated for Next-generation sequencing (NGS) library using NEXTflex Rapid DNA-Seq kit for Illumina (BIOO SCIENTIFIC, USA) following manufacturer’s recommendations. The library quality was quantified by Qubit dsDNA HS Assay Kit with the Qubit 2.0 fluorometer system (Invitrogen, Life Technologies, Grand Island, NY, USA). The multiplexed amplicons were sequenced using the Illumina HiSeq. 2500 platform to generate 250 bp paired-end reads. The sequencing and analysis were performed at SinoGenoMax Co., Ltd, Beijing, China.

### Microbiome bioinformatics

Firstly, we got high quality reads through filtering the original paired-end reads, then obtained longer sequences by splicing sequence, compared longer sequences with the 16 S reference database, removed chimeric sequences. Eventually filtered sequences were classified according to a certain threshold. Multiple sequence clustering operational taxonomic units (OTUs) was obtained at the end.

Representative sequences were taxonomically annotated using QIIME’s RDP Classifier^57^ against the Greengenes reference database. Alpha and beta diversity analyses were performed using QIIME’s core_diversity_analyses.py. For beta diversity analysis, the similarities between samples were calculated using Unweighted Unifrac^58^. Functional analyses were performed using PICRUSt software^59^ (a computational approach to predict the functional composition of a metagenome using marker gene data and a database of reference genomes).

### Construction of the HMDN including ASD

Based on the human microbe–disease association database (HMDAD)(http://www.cuilab.cn/hmdad), we calculated the microbe similarity between ASD and other diseases and constructed a network including ASD^34^.

### Statistical analysis

The Fisher’s exact test, randomization test and Wilcoxon signed-rank test analysis were performed using R (http://cran.r-project.org/). All p values reported in the study were from two-tailed tests and p values lower than 0.05 were accepted as significant in clinical data analysis. All p values for bacterial microbiome analyses were corrected using the Benjamini-Hochberg false discovery rate (FDR) correction, and the resulting corrected values were referred to as q values. q values lower than 0.05 were accepted as significant.

## Electronic supplementary material

Supplementary Figures

supporting dataset S1

supporting dataset S2

## Data Availability

All data generated during or analyzed during the current study are available from the corresponding author on reasonable request.
