# Supplementary material for: Analysis of gut microbiota profiles and microbe-disease associations in children with autism spectrum disorders in China
Source: Sci Rep. 2018 Sep 18;8:13981. doi: 10.1038/s41598-018-32219-2 (PMC6143520; doi:10.1038/s41598-018-32219-2)
Supplement: Supplementary file 1 — Supplementary Figures [file 41598_2018_32219_MOESM1_ESM.pdf]

Analysis of gut microbiota profiles and microbe-disease associations in children with autism spectrum disorders in China

Mengxiang Zhang, Wei Ma, Juan Wang, Juan Zhang, Yi He

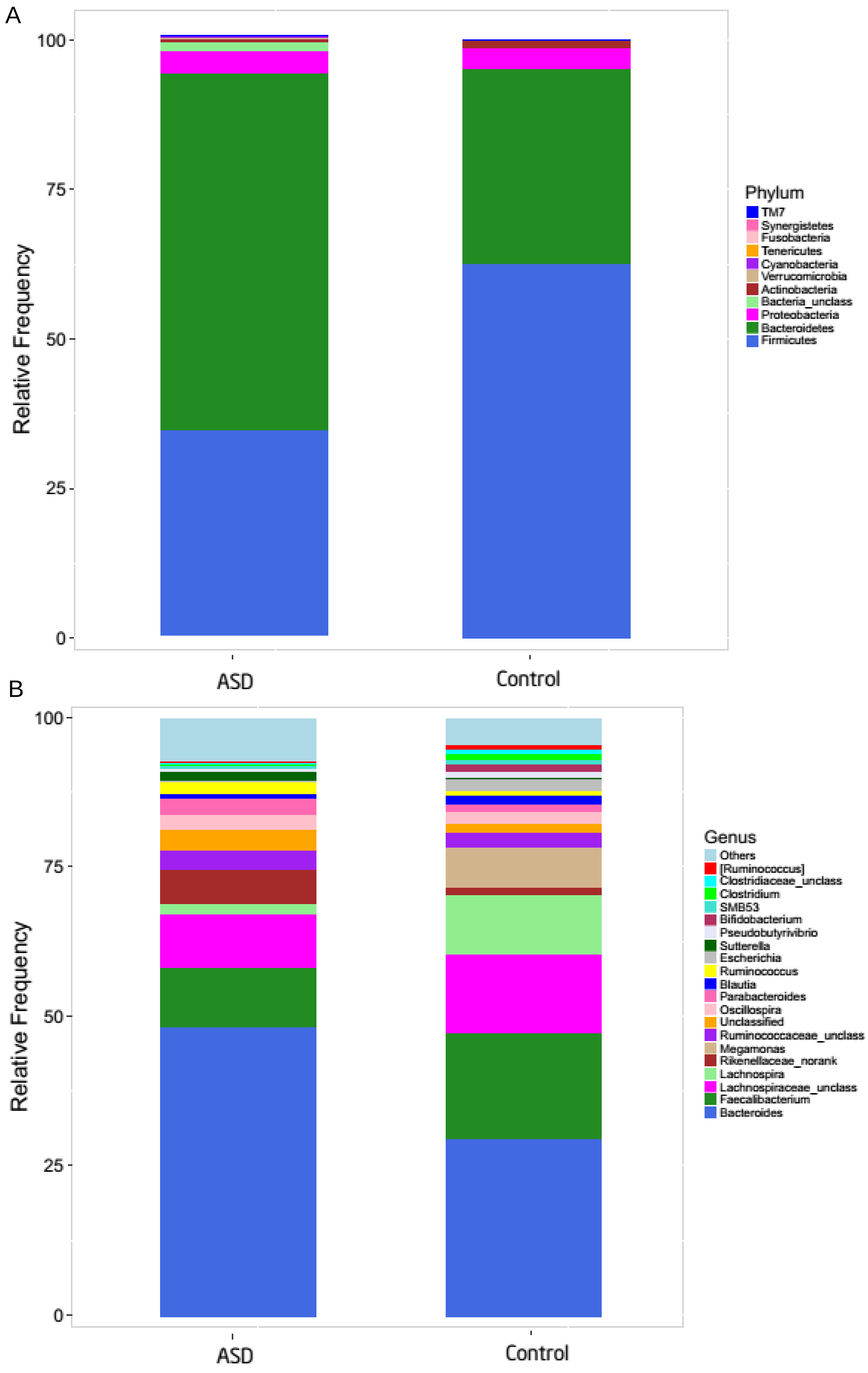
A
Relative Frequency
0
25
50
75
100
ASD
Control
Phylum
TM7
Synergistetes
Fusobacteria
Tenericutes
Cyanobacteria
Verrucomicrobia
Actinobacteria
Bacteria_unclass
Proteobacteria
Bacteroidetes
Firmicutes
B
Relative Frequency
0
25
50
75
100
ASD
Control
Genus
Others
[Ruminococcus]
Clostridiaceae_unclass
Clostridium
SMB53
Bifidobacterium
Pseudobutyrivibrio
Sutterella
Escherichia
Ruminococcus
Blautia
Parabacteroides
Oscillospira
Unclassified
Ruminococcaceae_unclass
Megamonas
Rikenellaceae_norank
Lachnospira
Lachnospiraceae_unclass
Faecalibacterium
Bacteroides

Figure S1. **A.** Microbial composition at phylum level. Compared to healthy controls, children with ASD had a significantly higher abundance of *Bacteroidetes*; **B.** Summary of bacterial genera detected in the two groups.

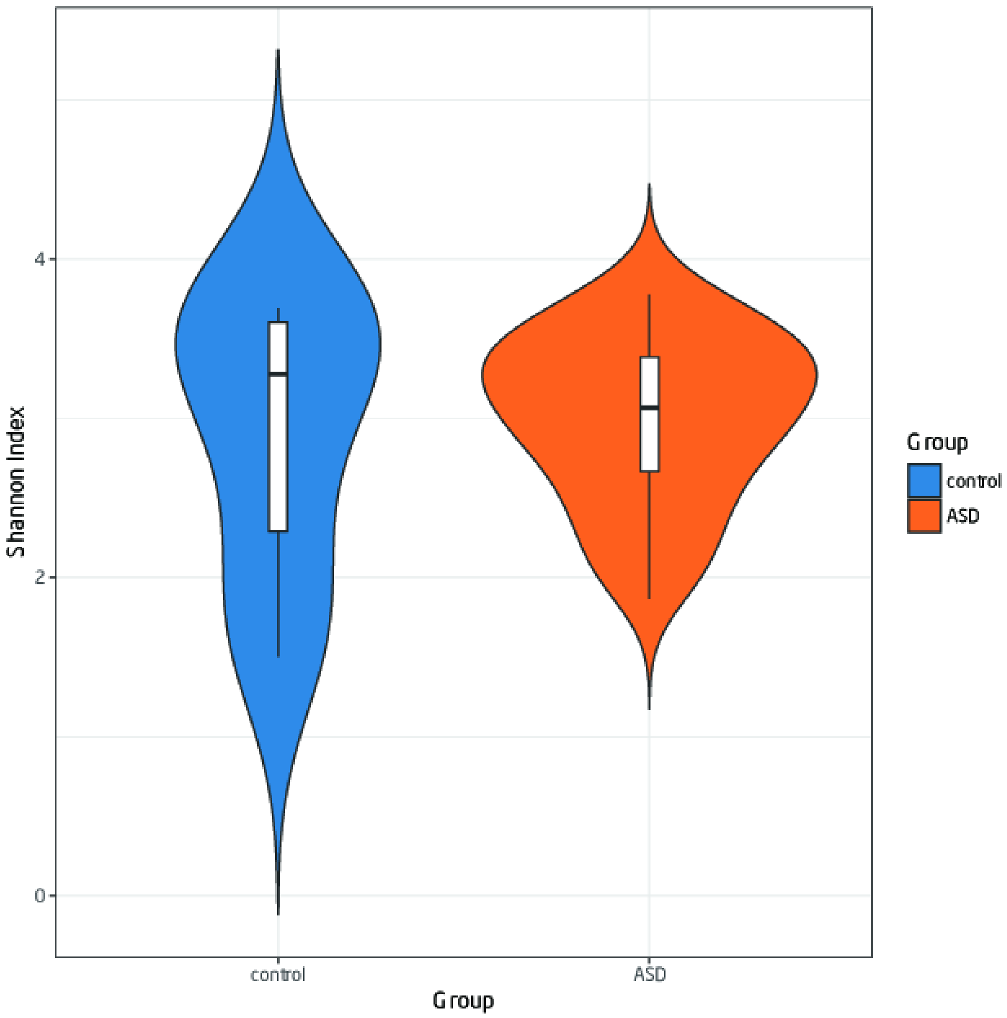

Figure S2. Comparison of Alpha diversity between ASD group and control group based on Shannon index. There is no significant difference in diversity between two groups.
